# Book Review

**Published:** 1914-03

**Authors:** 


**An International System of Ophthalmic Practice.** Edited by WALTER L. PYLE, A.M., M.D. **Therapeutics.** By Dr. A. DARIER, Paris. Translated by SYDNEY STEPHENSON, M.B., F.R.C.S. Pp. xiv, 444. London: Rebman Limited. 1911. 17s. 6d. net. —This book is divided into general and special therapeutics. The first half is a dictionary of all the remedies which have ever been used by anyone in eye work either as applied locally or for systemic use. As a work of reference it is too short in detail, while no one will willingly read through it for the purpose of acquiring knowledge. The second half is a dictionary of the diseases of the eye, followed in each case by suggestions for treatment. These are culled from all over the world, and as a result they are very unconvincing. The book had to be written to fill up the place in the series, and as such the best effort possible has been made. The author has been at pains not to overlook anything, and it will be found useful in suggesting treatment in cases which have not yielded to ordinary remedies.

**Salvarsan in Syphilis and Allied Diseases.** By J. E. R. MCDONAGH, F.R.C.S. Pp. vi, 164. London: Oxford Medical Publications. 1912. Price 7s. 6d.—Mr. McDonagh has given a most readable account of the administration of Salvarsan. His description of the technique is admirably clear and instructive, and he writes with commendable cautiousness of the wonderful results which follow the treatment. Chapter xvi is devoted to a discussion of the merits and use of Neo-Salvarsan. The book is indispensable to anyone who essays to treat syphilis according to the modern methods of Ehrlich.

**Diseases of Women.** By G. E. HERMAN, M.B., F.R.C.P., F.R.C.S., and R. DRUMMOND MAXWELL, M.D., F.R.C.S. 4th Edition. Pp. xiv. 899. London: Cassell & Co. Ltd. 1913. 25s. —We welcome another edition of Herman's well-known work on *Diseases of Women.* It is, of course, essentially the same as its predecessors, and is written in Dr. Herman's usual dogmatic style, with the quaint turns of phraseology every now and then which give such a charm to the book and help to fix the author's teaching in the reader's mind; but it has been brought in all parts thoroughly up to date, and in the matter of description of operative procedures is greatly in advance of previous editions. Herman's *Diseases of Women* has always held a position of its own amongst books on this subject. Its arrangement, based on the consideration of the main symptoms on account of which a patient may seek advice, marks it off at once from other books which aim at a systematic classification of some kind, and this peculiarity runs through the whole of the author's treatment of his subject. The aim of the work is, as the title states, to help in the investigation and treatment of any particular case rather than to present a clear view of the subject from the academical standpoint. The result is that the book is excellent for the practitioner to turn to for reference, full of wise suggestions and helpful advice drawn from much clinical experience, but is rather bewildering to the student, who needs his subject to be analysed for him according to some obvious scheme to assist him in memorising it for examination purposes. More attention might have been given to the pathological side, and the value of the book enhanced by the inclusion of good micro-photographs of morbid tissues. Some of the coloured plates are good, but in some the colouring is not so successful. The volume altogether is handsomely produced, and is much more elegant in arrangement, type, paper and binding than earlier editions. It is a book which could ill be spared, and we are glad to see it entering in the present form on a new lease of life.

**Diseases of the Eye.** By M. STEPHEN MAYOU. Second Edition. Pp. xiv, 306. London: Oxford Medical Publications. [1912.] 5s. net.—This is an excellent book in its way. It is packed full of information, but difficult to digest, for the student is not made to understand fully the why and wherefore of the various statements. Such knowledge is almost certain to be short lived. In the appendix is given a list of the various drops and ointments used in ophthalmic practice, also the visual requirements of the public services that have eye tests.

**Diseases of the Eye.** By Sir HENRY R. SWANZY, A.M., M.D., and LOUIS WERNER, M.B., F.R.C.S.I. Tenth Edition. Pp. xviii, 634. London: H. K. Lewis. 1912. 12s. 6d. net.—For many years this book has held its own as a great favourite both with students and practitioners. Considerable changes have been made in this edition, and the book has also a more convenient shape. Coloured figures have been introduced, and much up-to-date matter appears for the first time. There is so much to be compressed into a book of this sort, that some subjects suffer a little; for instance, the treatment of heterophoria only occupies half a page. This subject has become increasingly important of recent years, and anyone interested in it would naturally consult a special work. There are very few text-books which have condensed a subject so well as this one with so little detraction from its usefulness, and at the same time maintaining its readability.

**Mind and its Disorders.** By W. H. B. STODDART, M.D. Second Edition. Pp. xvi. 518. London: H. K. Lewis. 1912. 12s. 6d. net.—It was to be expected that a further issue of this book would be required at the end of a few years. One better designed and executed for its purpose could hardly be conceived. It is clear, concise and comprehensive; vexed questions are so far as possible avoided, and the reader may well commit to memory all presented to him in the assurance that he will not have soon to unlearn. The work is no mere compendium of the world's literature on its subject, but manifests the author's independent thought and research throughout, and, let us add, in an unobtrusive way. This thorough, general, personal investigation of all phases of insanity is responsible for the equable treatment of all of them, for the satisfactory scheme adopted, and the correct proportion between its sections. Most recent scientific research appears to have been under review, and what it is well to bring to the student's notice he will surely find in a clear and concise form. Thus, the psycho-pathology of Freud, the investigation of sub-conscious states in relation to his theories and to those of hysteria, the subjects of regional analgesias and areas of over-representation resulting, the thalamic basis of emotions, and many other matters of interest are set forth. The author's ability for clinical instruction is nowhere better shown than in the chapter on Dementia Precox. His grouping of various dystrophic conditions under the syndrome of confusional insanity and the alliance of this with the toxic psychoses is, we are sure, sound and conducive to the better diagnosis, classification and treatment of insanity. The preliminary section on normal psychology is an especially valuable one, and written with a direct relation to the chapters on Morbid Psychology which follow. The closing remarks on nerve-tissue staining and examination of cerebro-spinal fluid are well included for the sake of completeness. We strongly commend the book, which is well printed and suitably illustrated.

**Anæsthetics and their Administration.** By Sir FREDERIC W. HEWITT, M.V.O., M.A., M.D. Cantab. Prepared with the assistance of HENRY ROBINSON, M.A., M.D. 4th Edition. Pp. xv. 676. London: Macmillan & Co. Ltd. 1912. 15s. net.—Owing to recent developments in the field of anæsthetics, Sir Frederic Hewitt has found it necessary to re-edit his well-known book on the subject; and in this, the fourth edition, we find that not only has every part been brought up to date with some of the chapters re-written, but two entirely new chapters have been added—one dealing with local anæsthesia, the other with the medico-legal aspects of surgical anæsthesia.

Many of the newer methods have not yet been practised long enough to call for conclusive criticism, but the author has not shirked from expressing his opinion on such methods as spinal analgesia, open ether, intravenous ether, and combination of alkaloids with general anæsthetics. Spinal and local analgesia have not received such generous treatment as have most of the other subjects; the various methods of producing them have not been fully compared and discussed, and their description lacks almost all detail of technique. The chapter on the medico-legal aspects of anæsthesia, written by Sir Frederic Hewitt and Mr. Cotes-Preedy, could not have been expressed more lucidly or with greater fairness.

We feel that in producing this edition of his book the author has presented us with much temperate criticism concerning the multitude of recent innovations, each of which has in its turn been so confidently advocated by its particular supporters; and it is especially in this connection that the present edition may be relied on to afford help to the bewildered worker and warning to the enthusiastic experimenter.

**The "Nauheim" Treatment of Diseases of the Heart and Circulation.** By Leslie Thorne Thorne, M.D., B.S. Fourth Edition. Pp. vii, 104. London: Baillière, Tindall and Cox. 1913. 3s. 6d. net.—It is evident that the profession, and possibly also the public, are interested in the Nauheim methods of treatment, for this little book has reached a fourth edition. It opens with an account of the baths and graduated exercises of which the treatment consists. This is followed by a summary of indications and contra-indications, and the book closes with accounts of cases in which striking benefit has been perceived as a result of the treatment. Among the changes for the better which are ascribed to the treatment is the disappearance of extra-systolic arrhythmia, a form of improvement which is often achieved by merely limiting the patient's exercise.

**The Mineral Waters of Vichy.** By Charles Cotar, M.D. Pp. viii. 208. London: H. K. Lewis. 1913. 4s. net.—A foreword by Dr. Vaughan Harley demands that the waters of Vichy shall be regarded not as a pleasant beverage, but as a serious method of treatment of disease. Hydrotherapy claims to be a modern science, and the author endeavours to deal with the subject from this standpoint. After giving the theories concerning the origin of the waters and their chemical, hygienic and gaseous properties, he reviews their physiological action and deduces their therapeutic indications. The cult of a fashionable watering-place is sometimes founded on a "fashionable infatuation"; the hydrologist must be to a great extent guided by empiricism, and he must of necessity "glorify his office," but the fame of Vichy is beyond all criticism, and Dr. Cotar and his colleagues have an easy task in guiding many thousands of visitors yearly into better health by means of the never-failing artesian springs of the valley of the Allier.

**Laws of Sexual Philosophy.** By J. L. Chundra, L.M.S. Pp. v, 208. Calcutta. 1913. 4s. net.—It is perhaps unfair to criticise this book from a purely western standpoint, certainly it does not appear suited to the needs of the western student of gynæcology and obstetrics, for whom (in part) the author has apparently designed it. It is dedicated to a mythical lady apparently domiciled in the crescent moon, who bears a strong resemblance to the houris who adorn pictorial posters, and who owe their loveliness to somebody's soap, or somebody else's health food. The pages are filled with quotations from poets and philosophers, extracts from Aryan literature, and short accounts of the physiology and pathology of the genitalia. The quotations are not always exact; for instance, we noted some well-known lines of Tennyson attributed to Cowper, and the physiology is sometimes expressed in a curiously florid manner, which does not add to its scientific accuracy. The ethical position of the author is unimpeachable throughout, and for this reason we do not wish to criticise in further detail a book which is only likely to be used in this country for purposes for which it was never intended.

**Index-Catalogue of the Library of the Surgeon-General's Office, United States Army.** Second Series. Suaheli–Testut. Vol. XVII. Pp. 788. Washington. 1912.—We again bear testimony to the careful and arduous labours of the compilers of this indispensable series of references to medical literature. The present volume contains 2,357 author titles, 3,571 subject titles of separate books, and 36,898 titles of articles in periodicals. Syphilography takes up more than two hundred pages, and "surgery" is credited with more than a hundred. We always look forward with pleasure to the receipt of these annual volumes, for which we have no other words than those of praise.

**Transactions of the College of Physicians of Philadelphia.** Third Series. Vol. XXXIV. Philadelphia: Printed for the College. 1912.—We have not space to do more than briefly call the attention of our readers to these *Transactions*, which embody much good and interesting work. The present volume fully maintains their customary high level. We should, perhaps, especially notice the series of papers on epilepsy, the issue of a debate introduced by Dr. S. Weir Mitchell.

**The Medical Annual.** Bristol: John Wright & Sons Ltd. 1913.—The thirty-first annual issue maintains its character. The list of authors and contributors is somewhat smaller, and the use of a thin paper has reduced the bulk and weight to a minimum. We find facing page 1 the commencement of a glossary of new terms, and it is intended to make yearly additions to the list. The volume is well illustrated as heretofore, and it fulfils the aim of the publishers to provide the reader with the information he requires and to obtain it from the best source. The only way to review it is to keep it close at hand and consult it on every possible occasion. It rarely fails to give a satisfactory answer to an inquirer.

**Medical Laboratory Methods and Tests.** By HERBERT FRENCH, M.A., M.D. Third Edition. Pp. viii, 202. London: Baillière, Tindall & Cox. 1912. 5s. net.—This book has deservedly run into a third edition, and the author has taken the opportunity to revise, alter and make such additions as progress and experience have demanded. The methods are all good, and set out in such a manner as to make them easy to apply. It is a most commendable *vade mecum* for the practitioner possessing a medical laboratory.

**Lectures on Tuberculosis for Nurses.** By OLLIVER BRUCE, M.R.C.S., L.R.C.P. Pp. viii, 134. London: H. K. Lewis. 1913. Price 2s. 6d. net.—This book consists of six lectures delivered to the Queen Victoria Jubilee Nurses. It contains a very full account of the ætiology, diagnosis, treatment, prognosis and prevention of pulmonary tuberculosis, and is written in a clear and readable way, though it would not be an easy book for nurses who have no previous knowledge of medicine, pathology and bacteriology to help them. Thus there are descriptions of the staining reactions of the bacillus, of the theory of opsonins, and of the various anaphylactic tests which have recently been employed. Still, it is quite a good book for senior nurses who intend to take up tuberculosis work, and who have already a good knowledge of the general principles of medicine.

**Sciatica.** By WILLIAM BRUCE, M.A., LL.D., M.D. Pp. xi, 175. London: Baillière, Tindall & Cox. 1913. Price 5s. net. —In this little book Dr. Bruce elaborates the view that he has propounded before, that "Sciatica" is the result of reflex irritation originating from troubles in the hip-joint. It is illustrated by eighteen plates and radiograms, and an appendix contains very brief notes of seven hundred cases under the personal observation of the author. The book must be read in order to appreciate properly the author's position, and as it is short this will not involve a great expenditure of time. We confess that the chapter on treatment was to some extent a disappointment to us, as it does not seem to put forward anything particularly new or original; still, as the embodiment of the large experience of a careful physician who has given much attention to the subject it is to be received with respect.

**Minor Surgery.** By LEONARD A. BIDWELL, F.R.C.S. Second Edition. Pp. xvi, 299. London: University of London Press. 1912. 10s. 6d. net.—The fact that a second edition of this work has been required within twelve months indicates that it has been well appreciated, and in our opinion it deserves the appreciation it has received. The author's style is direct, lucid, and interesting. He has covered the ground of minor surgery judiciously and sufficiently, and we do not know of any book on the subject we can more confidently recommend to the student and practitioner.

**The Treatment of Hæmorrhoids and Rectal Prolapse by means of Interstitial Injections.** By DUDLEY D'A. WRIGHT, F.R.C.S. Pp. 20. London: Henry J. Glaisher. 1913. 1s. net. —In the preface to this pamphlet the writer affirms his belief, based upon long practice and observation, that there are few, if any, cases of hæmorrhoids which cannot be cured by interstitial injections. The great advantages of the treatment are that it can be carried out with a minimum of discomfort to the patient, and can be administered "from beginning to end without the necessity of the patient lying up or having the daily routine of work interfered with in any way." Detailed descriptions of the best methods of making the injections are given in the pamphlet, and it is our opinion that the treatment advocated is worthy of a much more extended application than it has hitherto received in this country.

**Pye's Elementary Bandaging and Surgical Dressing.** Revised and partly rewritten by W. H. CLAYTON-GREENE, M.B., B.C. Cantab., and V. ZACHARY COPE, M.D., M.S. Lond. Thirteenth Edition. Pp. viii, 230. Bristol: John Wright & Sons Ltd. 1913. 2s.—This well-known book, of size suitable for the pocket, can be strongly recommended to students beginning work in ward or casualty room, to surgical nurses, and to those studying first-aid in ambulance classes. It contains clear instructions, well illustrated, on all ordinary forms of bandaging, splinting, and wound dressing, and sections on the first treatment of syncope, shock, fits, drowning and poisoning. Directions for surgical case-taking are also given.

**Hygiene and Public Health.** By LOUIS C. PARKES, M.D., D.P.H. Lond., and HENRY R. KENWOOD, M.B., F.R.S. Edin. Fifth Edition. Pp. xi. 736. London: H. K. Lewis. 1913. 12s. 6d. net.—The fourth edition of this well-known textbook was reviewed in the *Journal* in December, 1911. We observe that it has added forty five pages to its already considerable bulk. There is little need to comment on its contents, which have been brought well up to date, and include much new matter. Its usefulness and popularity are well evidenced by the rate at which new editions appear, in spite of the competition with numerous other works of a similar character. We can confidently say that there is no better book on Hygiene and Public Health published in this country.

**Materia Medica and Pharmacy.** By REGINALD R. BENNETT, B.Sc., F.I.C. Second Edition. Pp. xx, 227. London: H. K. Lewis. 1912.—This is a pocket text-book specially compiled for medical students by the pharmacist to University College

Hospital. The classification adopted is one based on pharmacological action, and this fact alone renders the book superior to many of the treatises on the market. There is an important section on standardised preparations, and a summary of incompatibles. As a useful manual for examination purposes, and for subsequent reference, we can heartily recommend the book.

**Pathology of the Eye.** By P. H. ADAMS, M.B., D.O. Oxon. Pp. x, 194. London: Oxford Medical Publications. 1912. 5s. net.—This work is primarily intended for those preparing for special examinations in ophthalmology. It commences with a short account of the practical methods for sectioning and staining of specimens advised by the author. The bulk of the book is devoted to descriptions of the pathological conditions met with in eye diseases. This is clearly presented, incorporates the most recent views, and is illustrated by a series of excellent plates. At the end is given a short account of the bacteriology of eye diseases. While welcoming this work as giving in convenient form the present state of knowledge of ophthalmic pathology, we think that the portions devoted to laboratory work are not sufficiently full to serve as a practical guide in the laboratory.

**Bakteriologiske Studier over Barnetuberkulose.** By ARENT DE BESCHE, Ammanuensis ved det Pathologiska-Anatomicke Institut. Pp. 255. Kristiania: Steen'ske Bogtrykkeri. 1912. —Besche has examined lymphatic material obtained from autopsies upon 134 Norwegians. In each case he investigated the mesenteric, bronchial and cervical glands. Tuberculosis was present in 52 cases (38.8 per cent.). Tuberculosis was the cause of death in 28 cases. In 14 others tuberculosis was evident at the necropsy. In the remainder there was latent tuberculosis of the lymphatic glands only. Human tubercle bacilli were found present in 45 cases, and bovine tubercle bacilli in 3 cases. The pathways of infection appeared to be abdominal in 5 cases of the human type, and in 1 of the bovine group. In 5 other cases there were good grounds for assigning the portal of entry to the cervical glands. Besche concludes that the chief means of combating tuberculosis in children is the prevention of transmission of the bacteria from human carriers.

**Sclero-Corneal Trephining in the Operative Treatment of Glaucoma.** By Lieut.-Col. ROBERT HENRY ELLIOT, I.M.S., M.D., B.S. Pp. xviii. 117. London: George Pulman & Sons Limited. [N.D.] 7s. 6d. net.—This book covers the whole history of operations for glaucoma. It is, of course, written chiefly on the subject of the latest operation, namely sclero-corneal trephining. Col. Elliot is most enthusiastic about this operation. We feel he is quite right, and that it is the only operation for glaucoma, at any rate of the chronic kind. We also go so far as to think it is the best operation for the acute variety also. He has done his work at the Madras Ophthalmic Hospital, and has operated on 900 cases, so he must be a good authority on the subject. This book gives his experience of the operation, which he describes down to the veriest detail. We can thoroughly recommend a study of the work to anyone who intends to practise ophthalmology, and who up to now has looked on glaucoma as the bugbear of the ophthalmic surgeon.

**Annual Report of the Board of Regents of the Smithsonian Institution, 1911.**—Amongst the articles on all kinds of scientific subjects, from Sir Wm. Ramsay's presidential address to the British Association, down to an essay on Chinese architecture, which form the appendix to this volume of the Smithsonian Institute's Reports, there is one that is likely to be of supreme importance, at any rate to surgeons, viz. an article by Dr. Courmont, of Lyons, on the " Sterilisation of water by ultra-violet radiations." He passes water over a mercury-vapour lamp, and provided the water is translucent, the water is sterilised by a direct bactericidal action of the rays as fast as it flows over the lamp, and without altering the chemical composition of the water in the least. The author quotes Miguel, who tested the power of the rays in destroying spores. Miguel polluted water with Bacillus mesentericus ruber, " whose refractory spores will resist the temperature of boiling water sustained for several hours. With water thus polluted with 128,000,000 bacilli per liter, sterilisation is almost immediate (in the time necessary for the water to pass through the apparatus at the mean rate of 81 liters per hour). The author very truly says " this experiment should be of special interest to surgeons." Other papers of medical interest amongst this collection of scientific monographs are papers on the " Physiology of Sleep," " Factory Sanitation," and the " Physiological Action of Ozone." The volume is as usual profusely illustrated with most excellent reproductions of photographs.

**Studies in Clinical Medicine.** By C. O. Hawthorne, M.D. Pp. viii, 441. London: John Bale, Sons & Danielsson Ltd. 1912. Price 6s. net.—In this volume the author has published a collection of essays and lectures most of which have appeared in various journals. They were well worthy of being preserved in book form. Dr. Hawthorne has the gift of accurate observation and clear description, so that his essays are full of information conveyed with no mean literary grace. One of his best essays is the first, on " Rheumatism, Rheumatoid Arthritis and Subcutaneous Nodules." We like, too, the article on " The Authority of the *Pharmacopœia*," which embodies many excellent hints on the profitable use of this nowadays rather neglected publication. There are some interesting records of "cases illustrating recovery from symptoms of brain tumour." But the whole collection is full of examples of Dr. Hawthorne's fine clinical acumen.

**Modern Views upon the Significance of Skin Eruptions.** By H. G. Adamson, M.D., F.R.C.P. Pp. 103. London: John Bale, Sons & Danielsson Ltd. 1912. Price 3s. 6d. net.—In the Goulstonian Lectures for 1912 which are here published, Dr. Adamson emphasizes the changed point of view from which skin eruptions are regarded in modern times as contrasted with the older methods. "Now," as he rightly says, "if we name a new disease we try to indicate its origin and nature," whereas "the earlier names . . . described the appearance or some clinical character of the eruption." The series of lectures form a valuable epitome of the study of dermatology from the pathological aspect, and signalise a further breaking away from the "pictorial" dermatology of the nineteenth century which smothered itself with a cloud of ridiculous nomenclature.

**Manual of Surgery.** By Alexis Thomson and Alexander Miles. Vol. III.—**Operative Surgery.** Second Edition. Pp. xvi, 620. London: Henry Frowde and Hodder & Stoughton. 1913. Price 10s. 6d. net.—This volume, the second edition of the "Operative Surgery" part of Thomson and Miles' well-known work, has been thoroughly revised and brought up to date. It contains a complete review, necessarily somewhat sketchy owing to considerations of space, of modern operative technique. One cannot help thinking that the separation of the operative portion of what is essentially a student's System of Surgery from the rest is not free from objection. For instance, the volume under review contains a description of the operations for cleft palate, but one has to look elsewhere for any mention of the burning question of the date at which the operation should be performed. The amount of space allotted to various subjects is sometimes not well proportioned, the chapter on arteriorrhaphy being longer than that on hernia, and where space is necessarily at a premium, a description of so obsolete a procedure as Barker's subcutaneous suture of the patella might well be omitted. However, the volume as a whole is readable, sound and practical, and that it is appreciated by those for whom it is written is demonstrated by a second edition being called for so soon after the first.

**Coxa Vara: its Pathology and Treatment.** By R. C. Elmslie, M.S., F.R.C.S. Pp. 35. London: Oxford University Press. 1913. Price 2s. net.—The account of the pathology of coxa vara given in this pamphlet is based upon seventy-seven cases, of which thirty-three were adolescent, thirty-seven infantile, five congenital, and two aberrant, failing to fit into any group.

The pathological anatomy of the various forms of the disease was worked out by the author and published in the Erasmus Wilson Lecture in 1907. Further experience has confirmed the views there expressed and again recorded here. The illustrations are numerous and valuable, and the text clear and illuminating. All surgeons interested in the subject should study this exposition.

**Vaccine Therapy: its Theory and Practice.** By R. W. Allen, M.D., B.S. Fourth Edition. Pp. x, 444. London: H. K. Lewis. 1912. Price 9s. net.—This edition of a carefully written and useful manual contains about 170 pages more than its predecessor, in consequence of "the great developments and extensions which have been made in vaccine therapy" in the interval. After the preliminary chapters dealing with rationale and technique, the different parts of the body are taken in order, and the results to date of the various attempts that have been made to apply vaccine therapy to the disorders thus classified are detailed and commented on. The author is, as before, optimistic in his statements and anticipations, at the same time the material is presented to the reader in a clear and impartial manner, and the limitations and qualifications which stand in the way of unvarying success are duly insisted on. The two or three pages on asthma may be commended as an instance of this attitude; in fact, the whole chapter on the diseases of the respiratory tract is well worthy the perusal of practitioners, no matter what view they take of vaccine treatment. It is more or less a summary of the author's work on the *Bacterial Diseases of Respiration*, issued by the same publishers. In the section on eye diseases there is a remarkable case of cure of a case of gonococcal conjunctivitis with hypopyon (the prognosis of which had been pronounced hopeless) by a single large injection, given as a *dernier ressort*, of 250 million cocci, evidently from a stock vaccine, as the inoculation was done at the first visit. It is noteworthy that, as in most eye cases, it was a large dose that proved so efficacious. The question of dosage is still one of contradictions and mystery to the practitioner who appeals to the vaccine expert for advice, although we are accumulating results of experience which are empirically of great value. It may be said that vaccine treatment forced its way on the attention of the profession by sensational cases of the kind above quoted, effected by a few inoculations, and sometimes by one. And in fact the single dose which will at once effect a convincing improvement, and possibly give such a fillip that nature without further aid will accomplish the rest, is the great thing to aim at; and it must be confessed that at present there is no cut and dried method of arriving at it; above all, the patient himself and his condition at the moment should be carefully taken into account when deciding. As a rule, in vaccine treatment a first dose effects more improvement than any subsequent one, but there are many exceptions to this. Sometimes improvement is only maintained by continuing regular inoculations. Sometimes several inoculations will produce little effect, when suddenly after a fresh one, differing in no wise from its predecessors, the welcome " jump " of improvement takes place. The utility of tuberculin in cases of probably tubercular conjunctivitis is noted by the author, and the reviewer would be inclined to write still more strongly in its favour. The vexed question of the apparently opposed methods of giving tuberculin is gone into, and the present reviewer agrees entirely with the author's remark that " raised tolerance and diminished sensitiveness to tuberculin-toxin are desirable objectives, but they are not the real objective that we should have in mind." But this is a question that cannot be discussed in a brief notice. As regards the varieties of tuberculin, the author's remarks are equally sensible. Perhaps all that can be effected by tuberculin, and daily experience proves that it is a good deal, can be effected by judicious use according to the indications of old tuberculin, human or bovine T.R., or bacillary emulsion, or a mixture of human and bovine emulsions. Sensitised vaccines, a further development of therapeutics, are too recent to receive mention in this edition, and the same remark applies to " phylacogens." Considerable reserve is advisable in one's attitude towards these agents.

